# Investigating the Effects of Distillation System, Geographical Origin, and Aging Time on Aroma Characteristics in Brandy Using an Untargeted Metabonomic Approach

**DOI:** 10.3390/foods13121922

**Published:** 2024-06-18

**Authors:** Ruiqi Hu, Changqing Duan, Yibin Lan

**Affiliations:** 1 Center for Viticulture & Enology, College of Food Science and Nutritional Engineering, China Agricultural University, Beijing 100083, China; huruiqi@cau.edu.cn (R.H.); chqduan@cau.edu.cn (C.D.); 2 Key Laboratory of Viticulture and Enology, Ministry of Agriculture and Rural Affairs, Beijing 100083, China

**Keywords:** brandy, volatile profiles, untargeted metabonomics, GC-MS, PLS-DA

## Abstract

In this study, the influence of the distillation system, geographical origin, and aging time on the volatiles of brandy was investigated. An untargeted metabolomics approach was used to classify the volatile profiles of brandies based on the presence of different distillation systems and geographical origins. Through the predictive ability of PLS-DA models, it was found that higher alcohols, C13-norisopenoids, and furans could serve as key markers to discriminate between continuous stills and pot stills, and the contents of C6/C9 compounds, C13-norisoprenoids, and sesquiterpenoids were significantly affected by brandy origin. A network analysis illustrated that straight-chain fatty acid ethyl esters gradually accumulated during aging, and several higher alcohols, furfural, 5-methylfurfural, 4-ethylphenol, TDN, *β*-damascenone, naphthalene, styrene, and decanal were also positively correlated with aging time. This study provides effective methods for distinguishing brandies collected from different distillation systems and geographical origins and summarizes an overview of the changes in volatile compounds during the aging process.

## 1. Introduction

Brandy is a kind of aged alcoholic beverage, and grape brandy is a spirit drink produced by wine distillation that is matured in wooden casks. Its aroma characteristics and the presence of volatile compounds (esters, higher alcohols, acids, etc.) are mainly formed during fermentation, distillation, and the aging stage. Among them, the distillation process is a key process that affects the aroma quality of brandy [1]. During distillation, volatile compounds from grapes and fermentation will be transferred to the distilled wine through the evaporation and condensation of ethanol, forming unique aroma styles. The distillation process can maximize the content of alcohol and enhance aroma characteristics, reducing the occurrence of adverse aroma [2], which can not only concentrate the aroma substances produced by fermentation but also synthesize new aroma components through esterification reactions, synthesis reactions, maillard reactions, and dehydration reactions, increasing the complexity of flavors in grape spirits. The volatile compounds synthesized during the distillation process mainly include aldehydes, terpenes, norisoprenoids, and esters. Among them, furfural and aldehydes such as isobutyraldehyde and 2-methylbutanal are generated through Strecker degradation reactions, while *α*-terpineol, linalool, and other terpenes and norisoprenoids such as *β*-damascenone and 1,1,6-trimethyl-1,2-dihydronaphthalene (TDN) are generated by the degradation of their glycosides during distillation and heating [3].

**Figure 1:**
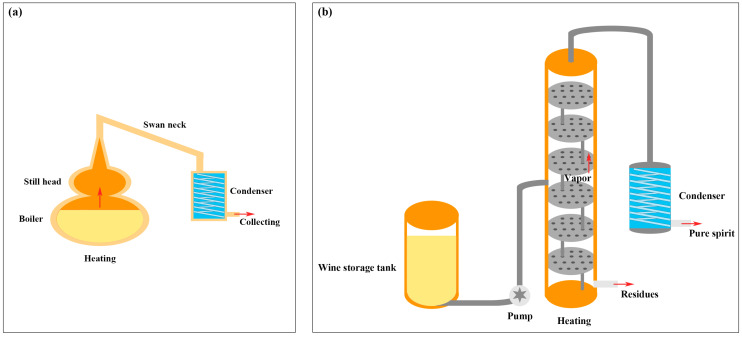
Schematic diagram of pot distillation (**a**) and continuous distillation (**b**).

Generally, the distillation process can be carried out by pot distillation or continuous distillation (Figure 1). The former one is known for the prestigious Cognac (France) produced by the Charentais Brandy pot still, which is a traditional batch process that is well described in terms of equipment and operation by the distillers and the relevant decree [3]. Charentais Brandy pot still equipment (Figure 1a) mainly consists of preheaters, distillers, condensers, manual or automatic control components, and the use of an open fire for direct heating or steam for indirect heating during distillation [4]. Pot distillation is a complex operation with a long distillation time and high energy consumption that requires cleaning during two-step distillation processes [5]. Pot distillation can produce more acetaldehyde, acetal, diethyl succinate, and ethyl acetate than continuous distillation, and the content of furanic compounds (furfural and 5-methyl furfural) is also higher [6], which can cause an unpleasant bitter almond flavor if it is excessive. Continuous distillation (Figure 1b) often uses a tower-type still with different types and quantities of trays depending on the separation requirements [7]. Compared to pot distillation, continuous distillation has a higher production efficiency, producing four times more esters and 20% more long-chain alcohols than pot distillation because of the increased flow rate and reduced heat intensity [8]. Therefore, winemakers will choose appropriate distillation methods based on the materials and wanted flavors.

On the other hand, a sensorial typicality can be observed between spirits according to the growing area (GA) of the grapes from which they originate [9]. Different origins can have a significant impact on methoxypyrazines, terpenoids, C13-norisioprenoids, and some esters due to the differences in climate, soils, and management practices that have led to the varied composition of the fruit and, consequently, the wines produced. The volatiles of Shiraz wines from two Australian geographical indications (GIs) were studied [10]. It was found that volatile compounds associated with wines from the cooler GI were grape-derived volatiles, such as monoterpenes, sesquiterpenes, green leaf volatiles, and some norisoprenoids. Therefore, the concentration of individual volatiles and their contribution to the total aroma of spirits were found to be associated with the botanical origin of the raw material [11]. The impact of barley variety and its geographical growth location on the flavor of newly produced spirits was also studied [12]. The results indicated that the environment and the interaction of variety and environment had a greater impact than variety alone.

Moreover, as the most time-consuming part of making brandy, aging plays an important role in remodeling the aroma style and will determine the quality of the brandy. Accordingly, brandy should be matured for at least 1 year or for at least 6 months in oak casks with a capacity of less than 1000 L [2], which can significantly modify the volatile compounds and sensory attributes of brandy, as these are influenced by many factors such as the oak species, the distillation system, and the aging time [13]. Since brandy is enriched by the extraction or release of phenolic and furfural compounds from the wood, the longer the aging time, the more the brandy is enriched. Compounds such as furfural, 5-methylfurfural, and 5-hydroxymethylfurfural, which contribute to the dried fruit flavor of brandy, are accumulated during aging [14].

Nowadays, volatile compounds in various beverages can be conveniently detected by gas chromatography–mass spectrometry (GC-MS). Moreover, it becomes more reliable and convenient to detect lower levels of compounds in samples by combining rapid techniques such as microextraction [15] or solid-phase microextraction (SPME) [16]. Using omics methods to discover multiple analytes in multi-omics studies is a promising approach to focusing on the overall analytes of objects toward a more detailed molecular understanding of analytes as well as the chain of cause and effect [17]. Foodomics is a combination of different related omics technologies presented as a global discipline in food, advanced analytical techniques, and bio-informatics. The molecular fingerprint of 120 commercial single-grape white wines (Chardonnay, Riesling, Sauvignon Blanc, and Silvaner) was investigated using the untargeted GC-MS method [18]. Compared with the 146 metabolites in 120 commercial white wines, the results suggested that the relative abundances of white wine metabolites (rhamnose, 4-hydroxybutyric acid, pyruvic acid, citramalic acid, malic acid, and galacturonic acid) could be markers for grape varieties.

In this study, untargeted headspace solid-phase microextraction gas chromatography–mass spectrometry (HS-SPME-GC-MS) was used to acquire the volatile profile of aged brandy samples from different distillation systems and geographical origins. Unsupervised (principal component analysis, PCA) and supervised (partial least-squares regression discriminant analysis, PLS-DA) multivariate analyses were used to investigate the effect of the distillation system and geographical origin on aroma compounds, and aroma compounds related to aging were studied by network analysis. The hypothesis of this study was that the distillation system, geographical origin, and aging time lead to varied volatiles in brandy, and those variations in volatiles could be used as potential markers to discriminate between different distillation systems, geographical origins, and aging times.

## 2. Materials and Methods

### 2.1. Chemicals

Analytical-grade NaCl was purchased from Beijing Chemical Works (Beijing, China). Chromatographic-grade ethanol was supplied by Honeywell Burdick & Jackson (Morristown, NJ, USA). Reference standards and C6-C24 n-alkanes were purchased from Sigma-Aldrich (St. Louis, MO, USA).

### 2.2. Brandy Samples

Thirty-six raw brandy samples were collected in Xinjiang. Among them, thirty-two samples were obtained using continuous distillation, and the remaining four samples were distilled in a pot still. Another group of brandies was collected from three different regions in China: 12 from Bohai Bay (Tianjin), 6 from Huaizhuo Basin (Shacheng), and 11 from Jiaodong Peninsula (Yantai), all of which were distilled from base wines made from Ugni Blanc (*Vitis vinifera* L.). Details of all the samples are given in Table S1.

### 2.3. HS-SPME-GC-MS Analysis

The extraction and quantification of volatiles in brandy were achieved by the combination of headspace solid-phase microextraction (HS-SPME) and gas chromatography–mass spectrometry (GC-MS) according to the method reported in a previous study [19]. Automated HS-SPME was performed using a CTC CombiPAL autosampler (CTC Analytics, Zwingen, Switzerland). Volatiles were extracted using a 2 cm DVB/CAR/PDMS 50/30 μm SPME fiber (Supelco, Bellefonte, PA, USA). A 5 mL sample of diluted brandy, obtained by adding deionized water to adjust the alcohol content to 14% (*v*/*v*), 1 g of NaCl, and 10 μL of 4-methyl-2-pentanol (internal standard, 1 g/L), was prepared in a 20 mL vial capped with a PTFE–silicone septum. The initial alcohol content in brandy was measured using an alcohol meter (CJM-091, Chuangjimei Instrument & Meter, Hengshui, China) at 20 °C. After equilibration at 40 °C for 30 min, volatile extraction was carried out by inserting a preconditioned SPME fiber into the headspace of the vial at 40 °C for 30 min while stirring at 500 rpm. The fiber was thermally desorbed by insertion into the injection port for 8 min in a splitless mode.

GC-MS analysis was performed on an Agilent 6890 GC coupled with an Agilent 5975C MS. An HP-INNOWAX capillary column (60 m × 0.25 mm × 0.25 μm, J&W Scientific, Folsom, CA, USA) was used for the chromatographic separation. The temperature of the injector was 250 °C. The carrier gas was helium at a constant flow rate of 1.0 mL/min. The initial oven temperature was held at 50 °C for 1 min, then increased to 220 °C at 3 °C/min and held for 5 min. The temperatures of the MSD transfer line, ion source, and quadrupole were set at 250 °C, 230 °C, and 150 °C, respectively. The mass spectrometer was operated in electron ionization (EI) mode at 70 eV in the full-scan mode (*m*/*z* 30-350).

### 2.4. Quantification of Volatile Compounds

The retention index (RI) of each volatile was calculated using the Automated Mass Spectral Deconvolution and Identification System (AMDIS). The identification of volatile compounds was achieved by comparing the obtained mass spectra and retention indices (RIs) with those of reference standards and compounds in the NIST 2014 mass spectrometry database. Using MSD ChemStation Data Analysis (Agilent Technologies, Inc., Santa Clara, CA, USA) to integrate the peak area, the ratio of each volatile to the internal standard peak area was used as the quantitative basis. Quantitative analysis was carried out using calibration curves of standards with different gradients prepared in a simulated liquor solution (14% *v*/*v* ethanol/water solution).

### 2.5. Data Pre-Processing and Statistical Analysis

#### 2.5.1. XCMS Online

The raw GC/MS data files were converted to ‘NetCDF’ files using the Agilent MSD ChemStation software (G1701FA, F.01.01.2317, Agilent Technologies, Inc.). The converted files were then imported into XCMS-Online ”https://xcmsonline.scripps.edu (accessed on 9 August 2019)” for feature detection, retention time correction, peak alignment, and grouping [20]. The XCMS centWave algorithm was used with the following parameters: ppm = 100, peakwidth = c (5, 10), mzdiff = 0.01, prefilter peaks = 3, prefilter intensity = 100, and noise = 0. Feature alignment was carried out with the following parameters: method = “density”, bw = 10, mzwid = 0.25, minfrac = 0.5, and minsamp = 1. Retention time correction was performed with the ‘obiwarp’ method with profStep = 1. The output table (exported as a .csv format file), including sample names, feature indexes (*m*/*z*, RT), and peak intensities, was exported for multivariate statistical analysis.

#### 2.5.2. Statistical Analysis

In order to classify the distillate or brandy samples according to distillation system and geographical region, principal component analysis (PCA) and partial least discriminant analysis (PLS-DA) were carried out to analyze the extracted features (intensity of a given *m*/*z* at a certain time) using MetaboAnalyst 3.0 “https://www.metaboanalyst.ca/ (accessed on 19 August 2019)”. Normalization was performed using ‘Autoscaling’ in the MetaboAnalyst 3.0 program (mean centered and divided by the standard deviation of each variable). Features were selected in PLS-DA according to variable importance in projection values (VIP > 1), and the corresponding volatiles were determined based on feature retention times and mass/charge ratios. When multiple features corresponded to a volatile, the feature with the highest VIP values was used to represent the volatile compound. After determining the characteristic volatiles through PLS-DA, the impact of various treatments on the volatiles was clarified through one-way ANOVA and cluster heat mapping. One-way analysis of variance (ANOVA), cluster heat mapping, and Pearson correlation evaluation were all performed using R 4.3.1. For one-way ANOVA, the normal distribution test of the data was completed using Shapiro’s test, the homogeneity of variance was completed using Bartlett’s test, and the function ‘aov’ provided the calculation and testing of the analysis of variance. Cluster heat mapping was performed using ‘pheatmap’ packages, and Pearson correlation evaluation was based on the ‘rcorr’ function in ‘Hmisc’ packages. The correlation network among the compounds during the aging process was visualized using Gephi version 0.10.1 software “https://gephi.org/ (accessed on 19 November 2023)”.

## 3. Results and Discussion

An integrated metabolomics workflow for data analysis was used, following Awale et al. [21]. Using XCMS online, we identified a total of 1085 and 905 metabolite features in different distillation systems and origins of brandies (Tables S2 and S3). A feature is a molecular entity with a definite mass and retention time, designated as M (mass/charge) and T (time in minutes) [22]. The qualitative and quantitative results of all the compounds from each treatment are listed in Tables S4 and S5.

### 3.1. Exploratory Data Analysis by Principal Component Analysis (PCA)

**Figure 2:**
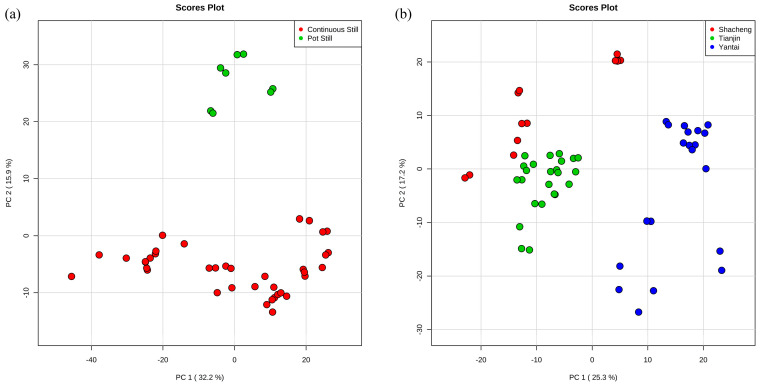
Multivariate statistical analysis of features from two distillation systems and three origins. Principal component analysis (PCA) scores plot in (**a**) distillation systems and (**b**) origins.

Unsupervised principal component analysis (PCA) was carried out on the features to explore the differences among the multivariate samples. The impact of the distillation systems on the brandy can be observed in the PCA scores plot, where 32.3% and 15.9% of the total variance was explained by PC1 and PC2, respectively (Figure 2a). It could be clearly seen that the second component differentiated between continuous still and pot still distillates, as pot still was all concentrated on the positive half of the y-axis (Figure 2a). For the different origins of brandies, PC1 explained 25.3% of the variance, while PC2 captured about 17.2% of the total variance (Figure 2b). PC1 separated Yantai from Shacheng and Tianjin, as Yantai was concentrated on the positive half of the x-axis. Although there was not a complete separation between Shacheng and Tianjin, it could still be observed that Shacheng was more concentrated on the positive half of the y-axis, while Tianjin was more concentrated on the negative half of the y-axis, indicating the different presence of metabolites between the two origins (Figure 2b).

### 3.2. Classification of Original Brandies According to Distillation Systems

**Figure 3:**
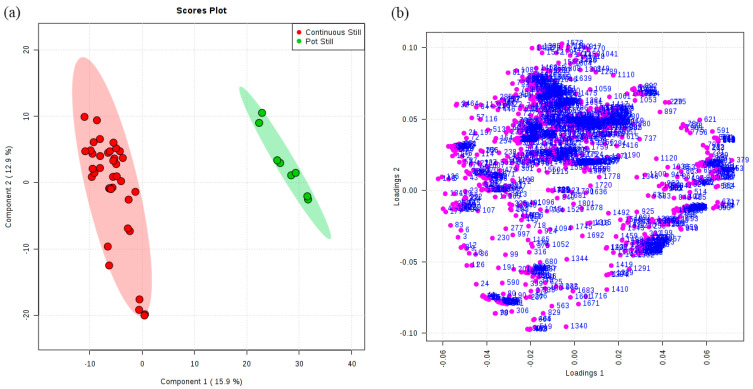
Partial least-squares-discriminant analysis (PLS-DA) scores plot (**a**) and loadings plot (**b**) for distribution of volatile features of distillation systems.

For a better understanding of the metabolic characteristics and interpretation of the results obtained by the unsupervised analysis model and to highlight the similarities and differences between treatments, the partial least-squares-discriminant analysis (PLS-DA) method was applied. The PLS-DA model according to distillation systems showed good predictive ability with Q^2^ = 0.94 and R^2^ = 0.90 (Table 1). Of the 1085 identified features that characterized the distillation systems, 354 were selected with VIP (variable importance in projection) values obtained from the PLS-DA model that were greater than 1, with 169 features expressing higher intensity in continuous still brandies, contrasting with 185 features expressing higher intensity in pot still brandies (Table 1). Pot still brandies were set up as one group, while continuous still brandies were set up as another group to investigate the effect of two different distillation systems on the volatile profile (Figure 3a). By matching the corresponding volatiles through the retention time, features (VIP > 1) were selected. If multiple features corresponded to the same volatile compound, the feature with the highest VIP value was used to represent the volatile compound. The volatiles screened in the above way are listed in Table 2. A total of 32 compounds were selected with VIP values of their features greater than 1 (Table 2), and 26 compounds were significantly different (*p* < 0.05) due to the distillation systems. Using the VIP values obtained from the PLS-DA model, it is possible to determine potential flavor markers in the selected classes. Combining the results of the one-way ANOVA, it was shown that pot still distillation made brandy dominant on furfural, *β*-damascenone, isoamyl acetate, hexyl acetate, vinyl decanoate, *α*-ionene, 2-phenethyl acetate, ethyl heptanoate, propane, 1,1,3-triethoxy-, ethyl butanoate, 1,1,6-trimethyl-1,2-dihydronaphthalene (TDN), linalool, and 1-decanol, all of which exhibited significantly higher content (*p* < 0.001) compared to continuous still distillation. The application of a continuous still confirmed a significantly higher content of 1,5-dimethylnaphthalene, isobutyl alcohol, 1-hexanol, 1-heptanol, methyl octanoate, ethyl heptanoate, ethyl nonanoate, ethyl salicylate, 1-nonanol, methyl decanoate, ethyl butyl succinate, (*S*)-3-ethyl-4-methylpentanol, diethyl succinate, and (*E*)-3-hexen-1-ol (Table 2).

**Figure 4:**
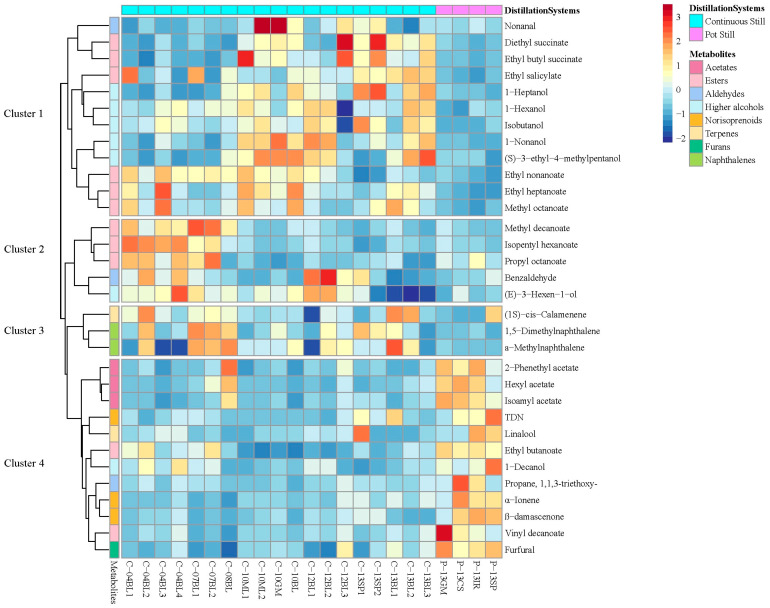
Cluster heat map of the abundance of brandy volatiles (VIP > 1) from distillation systems.

In order to concretely observe the influence of the two distillation systems on the brandy components, a cluster heat map was introduced (Figure 4). From the cluster heat map, we divided all the compounds into four clusters based on their concentrations in the two distillations. It could be found that the compounds with significantly higher content in pot still brandies, as found in one-way ANOVA, were concentrated in cluster 4, which contained all the furans (furfural) and norisoprenoids (*β*-damascenone, *α*-ionene, and TDN). Furfural, as a furan compound, can be affected by the distillation systems and oak wood type, according to other studies. Furfural and its derivative, 5-methylfurfural, are the most important class of furan compounds. They are oxygenated pentacyclic compounds that do not occur in nature and are usually the product of a thermal reaction [23]. In brandy, furans are usually generated during the distillation and the oak barrel storage stages [14]. It was found that a significantly higher proportion of furfural was obtained in pot still distillates (concentration: 0.584 μg/L) than in continuous still distillates (concentration: 0.141 μg/L) (Table 2). It is well known that the distillation time of pot stills is longer than that of continuous stills, which favors the synthesis of furan compounds through carbohydrate degradation [6], which was consistent with our study and manifested as furfural being significantly higher in pot still distillates than in continuous still distillates. Norisoprenoids were also found to be more abundant in pot still distillates. Norisoprenoids are important flavor compounds in wine and brandy; for instance, *β*-damascenone, which can impart floral and cooked fruit flavors to wine, and TDN, usually described as kerosene [24], are often formed by hydrolytic reactions that occur during distillation and are favored by high ethanol content and high temperatures [25]. Norisoprenoids were found to be generated during the first distillation step of Cognac wine spirit due to chemical reactions induced by high temperatures [3]. Compared to continuous stills, pot stills have a longer high-temperature distillation time, which is more conducive to the synthesis of norisoprenoids. This may be the reason why the content of norisoprenoids in pot still distillates was higher than in continuous still distillates. The above discussions suggest that furans and norisoprenoids could be considered potential markers of pot still distillation since all of them are abundant in pot still distillates due to their extended distillation time in a high-temperature environment, which provides sufficient time for synthesis.

As all higher alcohols were significantly (*p* < 0.05) more abundant in continuous stills (Table 2), all of them were gathered in cluster 1 except for (*E*)-3-hexen-1-ol (Figure 4). The concentration of higher alcohols in the two distillations was consistent with the previously reported results, as the original pot still brandies were composed mainly of the heart fraction, but higher alcohols were more abundant in the head fraction than in the heart fraction in pot still products due to the lower boiling point and higher solubility in ethanol than in water [26]. 1,5-Dimethylnaphthalene and α-methylnaphthalene are two naphthalenes identified in this study; they were clustered in cluster 3 and were more abundant in continuous still distillates than in pot still distillates (Figure 4). 1,5-Dimethylnaphthalene was the second most important compound in the PLS-DA models, with VIP values of 2.32, which meant that 1,5-dimethylnaphthalene was an important compound distinguishing pot stills and continuous stills. In wine, naphthalenes have usually been described as off-flavors with a chemical note [27]. The reason why naphthalenes were more abundant in continuous still distillates than in pot still distillates may be due to the difference in separation efficiency between these two distillation systems. Continuous distillation often uses tray columns; the function of the tray is to increase the contact area of the gas–liquid phase, provide heat and mass transfer, and thereby improve the separation efficiency [28]. Benefiting from higher separation efficiency compared to pot stills, continuous stills may make it easier to collect naphthalenes during the distillation process.

Most esters were clustered on cluster 1 and cluster 2, while all the acetates were gathered on cluster 4 (Figure 4), suggesting that the enrichment and loss of esters varied with different distillation systems, as acetates were easier to accumulate through pot stills, while medium-chain esters such as ethyl nonanoate, methyl octanoate, ethyl heptanoate, methyl decanoate, and isopentyl hexanoate were more abundant through column stills. The chain length and structure of esters largely determine their decomposition process at different distillation temperatures and their solubility in water/ethanol solutions [2]. In a pot still, the distilled liquid is a mixture of mainly water and alcohol, along with smaller amounts of other by-products of fermentation [5]. Due to the presence of azeotropes in the system, the corresponding azeotropic temperature of each ester also varies. In the C1-C6 alcohol/ester/water systems, as the carbon chain of esters and the complexity of their own structure increased, their azeotropic temperature also increased, suggesting that in this complex azeotropic system, short-chain esters were more easily collected by distillation than long-chain esters [29]. While in the continuous still distillation medium-chain ethyl esters (C6-C12) were more easily collected than short-chain ethyl esters (C1-C4) under certain feed rates as the temperature increased (from 40 °C to 120 °C), and at a feed rate of 100 g/h, when the distillation temperature rose to 100 °C, the yield of C4 ethyl esters in the distillate was 0%, while the C6, C8, and C10 ethyl esters still maintained a yield of 0.1%–0.2% [30]. Combined with our results, it could be found that short-chain esters were more likely to be collected through pot stills, while it was easier to collect medium-chain esters than short-chain esters in continuous stills.

### 3.3. Classification of Brandies According to Geographical Origin

**Figure 5:**
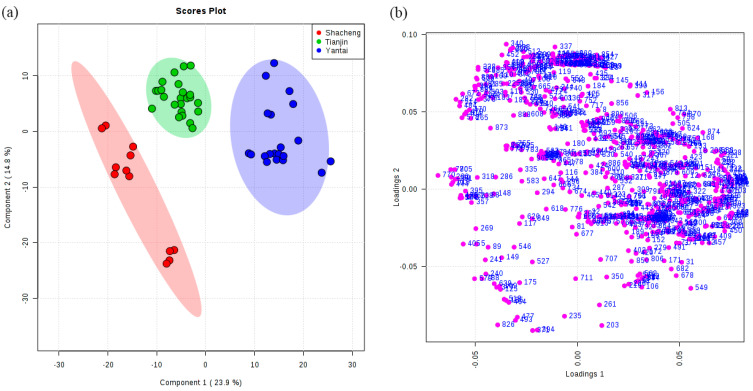
Partial least-squares-discriminant analysis (PLS-DA) scores plot (**a**) and loadings plot (**b**) for distribution of volatile features of origins.

The samples were collected from three different geographical origins, as follows: 6 from Shacheng, 12 from Tianjin, and 20 from Yantai. The PLS-DA models according to geographical origin showed good predictive ability with R^2^ = 0.85 and Q^2^ = 0.82 (Table 1). Out of the 905 features identified in brandies from three geographical origins, 235 were selected with VIP values greater than 1 (Table S3). The brandies collected from the three origins were set up as three groups in the PLS-DA model to investigate the effect of geographical origin on the volatile profiles (Figure 5a). The qualitative composition of compounds from the identified features was the same as that in Section 3.2. A total of 24 compounds with VIP values greater than 1 were selected, namely, 4 terpenes, 2 norisoprenoids, 4 higher alcohols, 2 aldehydes, 9 esters, and 3 acetates.

**Figure 6:**
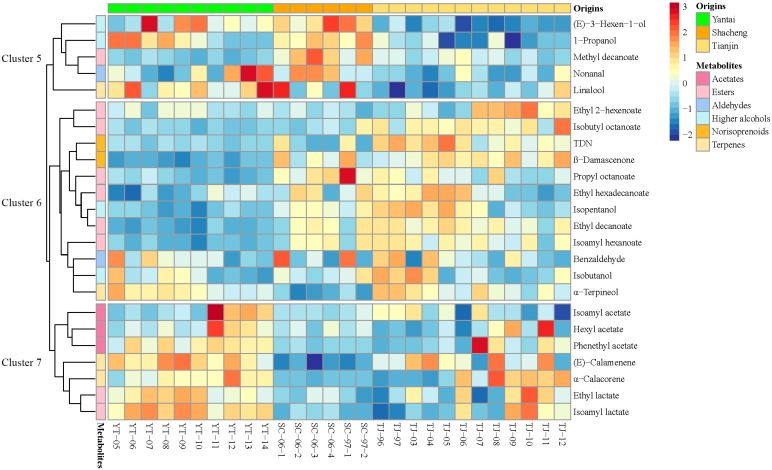
Cluster heat map of the abundance of brandy volatiles (VIP > 1) from distillation systems.

Among the above 24 compounds with VIP values greater than 1, 12 compounds were identified as having significant differences (*p* < 0.05) (Table 3). Through the results of one-way ANOVA, the volatile compounds of all three regions with VIP values greater than 1 were well differentiated (Table 3). From the results of one-way ANOVA alone, it could be found that the contents of methyl decanoate, 1-propanol, propyl octanoate, and (*E*)-3-hexen-1-ol in Shacheng brandies were significantly higher than in the others, and the contents of *α*-terpineol, *β*-damascenone, TDN, isobutyl octanoate, *α*-calacorene, ethyl 2-hexenoate, and ethyl decanoate in Tianjin brandies were significantly higher than in the others. As for Yantai, only ethyl lactate was significantly higher than in the other two (Table 3). To better understand the profile of the compounds in each sample, cluster heat mapping was applied, and the compounds were divided into three clusters based on the results. It could be found that the compounds in cluster 5 were more abundant in Shacheng and Yantai brandies, especially in Shacheng brandies; the compounds in cluster 6 were more abundant in Shacheng and Tianjin brandies; and the compounds in cluster 7 were more abundant in Yantai and Tianjin brandies (Figure 6).

From cluster 5, it was found that all samples from Shacheng and YT-07, YT-09, and YT-10 from Yantai were dominated by (*E*)-3-hexen-1-ol, and nonanal was abundant in SC-06-2, SC-06-3, and SC-06-04 from Shacheng and YT-12, YT-13, and YT-14 from Yantai. In addition, all samples from Tianjin had lower concentrations of (*E*)-3-hexen-1-ol and nonanal. Both (*E*)-3-hexen-1-ol and nonanal are C6/C9 compounds mainly generated through the lipoxygenase-hydroperoxide lyase (LOX-HPL) pathway, and their derivatives are prevalent in grapes and mainly contribute to the “herbaceous” and “green” aroma in wine and brandy [31]. C6/C9 compounds can also be regarded as indicators of immature grapes affected by a series of environmental factors that can promote grape ripening, such as excessive solar radiation, extended sunshine duration, and warm weather [32], all of which can reduce the content of C6/C9 compounds in grapes and, subsequently, in brandy. The different concentrations of C6/C9 compounds in the three regions might be caused by different irradiation conditions.

Cluster 6 contains five esters, two higher alcohols, two norisoprenoids, one aldehyde, and one terpenoid. It is worth noting that all the norisoprenoids (TDN and *β*-damascenone) were in cluster 6 and were mostly abundant in the samples from Tianjin. C13-norisoprenoids would be affected by the grape cultivation environment during the ripening process of grapes. With the increase in temperature and light exposure, the amount of C13-norisoprenoids would increase accordingly [13]. The different accumulations of norisoprenoids in the three regions might be caused by different photothermal resources.

Cluster 7 contains three acetates, two terpenoids, and two esters. (*E*)-calamenene, *α*-calacorene, ethyl lactate, and isoamyl lactate were abundant in most samples from Yantai and some samples from Tianjin. (*E*)-Calamenene and *α*-calacorene are a kind of sesquiterpenoid directly derived from grapes and exist in free forms after production [33]. The expression level of sesquiterpenoids is related to the process of grape ripening [33,34]; for example, (*E*)-calamenene and *α*-calacorene decrease with the maturation of Shiraz grapes [33]. Subsequently, the evolution of terpenes throughout different phenological stages from fruit set to harvest was studied [34], revealing that the concentrations of (*E*)-calamenene and *α*-calacorene would decrease during the maturation of grapes. Thus, the concentration of (*E*)-calamenene and *α*-calacorene in Yantai might be related to the maturity of grapes and their harvest time.

### 3.4. Network Analysis

**Figure 7:**
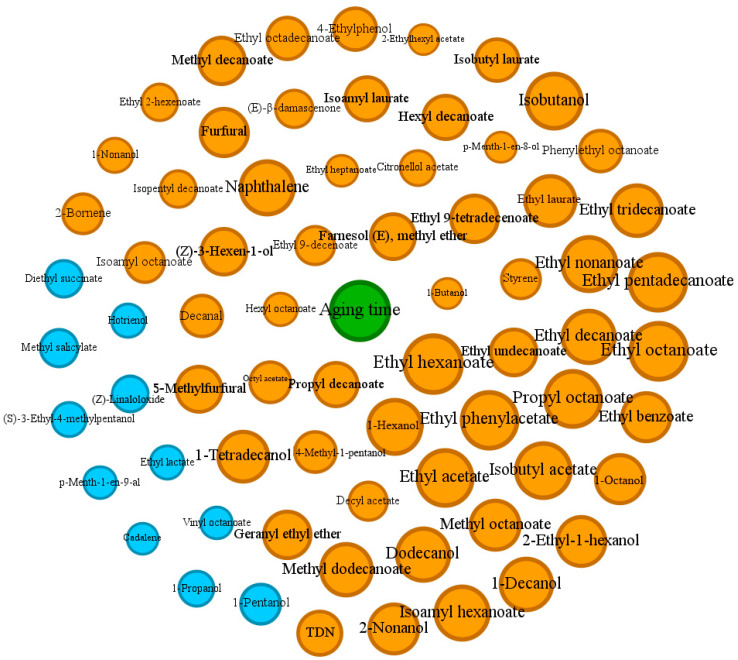
Network analysis of brandy volatiles (*p* < 0.05) and aging times from all samples. Orange circles and blue circles represent volatiles positively and negatively correlated with aging times, respectively. The diameter of circles and the font size reflect the extent of correlation.

In our study, all samples were aged for different years (Table S1). To explore the correlation among the compounds during the aging process, a Pearson correlation analysis was performed between the aroma compounds in each treatment and their corresponding aging times. A total of 69 compounds that significantly (*p* < 0.05) correlated with aging times from all the compounds in the above treatments were selected and visualized using network analysis (Figure 7). Among them, 58 compounds were positively correlated with aging times, and 11 compounds were negatively correlated. In the visualization of the correlation results using network analysis, the circle diameter reflects the absolute value of the correlation coefficient between the compounds and the aging times (Figure 7).

It was found that there were far more compounds positively correlated with aging times than negatively correlated compounds, and the negative correlation was not as strong as the positive correlation. From the network analysis, it could be directly observed that a large proportion of the compounds that positively correlated with aging times were esters, and there were actually 35 esters positively correlated with aging times. Most of the esters were formed through the alcoholysis of the corresponding acids, actively facilitated by yeast [35]. For example, ethyl esters of straight-chain fatty acids (e.g., ethyl hexanoate, ethyl octanoate, and ethyl decanoate) were mostly derived from the yeast lipid metabolism, and ethyl esters of branched short-chain fatty acids (e.g., ethyl isobutanoate, ethyl 2-methylbutanoate, and ethyl isopentanoate) were derived from the yeast amino acid metabolism. The hydrolysis and formation of esters during the aging process depend on many factors. During aging, the esterification and hydrolysis reactions are entirely reversible, so the factors that affect the reaction rate in one direction will also affect the reverse reaction similarly. The composition changes in the storage environment, such as the increase in free hydrogen ions along with a pH decrease or the presence of undissociated protons of organic acids, can all serve as catalysts for these reactions [36]. In our study, many ethyl esters of straight-chain fatty acids, such as ethyl octanoate, ethyl hexanoate, ethyl pentadecanoate, ethyl laurate, ethyl tridecanoate, and ethyl decanoate, were all positively correlated with aging times. It was suggested that during the aging process of brandy, the rate of esterification of these esters was greater than that of hydrolysis. This may be due to the high ethanol content of brandy, which inhibited the hydrolysis of the esters to some extent and thus favored the reaction to proceed toward esterification. Besides ethyl esters of straight-chain fatty acids, other esters of straight-chain fatty acids, such as methyl decanoate, methyl dodecanoate, hexyl decanoate, isoamyl laurate, and propyl octanoate, were also found to be positively correlated with aging times, indicating that a high alcohol environment would affect the evolution of esters of straight-chain fatty acids. Consistent with the results of previous studies, ethyl esters of fatty acids would increase with the brandy aging times of oak barrels [6], further demonstrating the authenticity of the accumulation of esters of straight-chain fatty acids during the brandy aging process.

Most of the higher alcohols were positively correlated with aging times, such as 1-decanol, 1-tetradecanol, 1-hexanol, isobutanol, 2-nonanol, 1-octanol, 4-methyl-1-pentanol, etc., while 1-pentanol, 1-propanol, and (*S*)-3-ethyl-4-methylpentanol were negatively correlated with aging times, suggesting that each higher alcohol accumulated and decomposed differently through aging. Compounds derived mostly or entirely from oak, such as furfural, 5-methylfurfural, and 4-ethylphenol, were positively correlated with aging times, which was consistent with previous research [14]. In addition, TDN, *β*-damascenone, naphthalene, styrene, and decanal were also positively correlated with aging times, suggesting that the concentrations of these compounds would increase with aging.

## 4. Conclusions

The untargeted metabonomic approach allowed us to search for spectral information from chromatograms without prior identification of the compounds. Using multiple statistical analysis methods, our study showed that brandies from different distillation systems and origins had significant differences in their aroma profiles. Based on the predictive ability of the PLS-DA model, we quantified the compounds for which features had VIP values greater than 1. This study found that 26 compounds could be used as characteristic compounds to distinguish brandies from pot stills and continuous stills, and 12 compounds could be used as important markers to distinguish brandies from different geographical origins. A network analysis was performed to explore the relationship between various compounds and aging time. It was further found that ethyl esters of straight-chain fatty acids would increase with the aging time of brandy in oak barrels.

## Figures and Tables

**Table 1:** PLS-DA models based on independent sample.

Model	Class	Numbers	Correct Classifications (%)	R^2^ (%)	Q^2^ (%)	Selected Variables (VIP > 1)	GC-MS Peaks
							(VIP > 1)
1	Continuous Still	38	100	0.94	0.9	354	32
	Pot Still	8	100				
2	Shacheng	12	100	0.85	0.82	235	24
	Tianjin	24	100				
	Yantai	20	100				

**Table 2:** Mean concentration (μg/L) and standard error of mean (SE mean) of brandy volatiles (VIP > 1) from pot still and continuous still.

Compound	Feature ID	VIP	Significance	Pot Still	Continuous Still
(*1S*)-*cis*-Calamenene ^3^	379	2.469	-	0.002 ± 0.001	0.001 ± 0.001
1,5-Dimethylnaphthalene ^3^	540	2.3174	**	0.000502 ± 0.000129	0.00121 ± 0.000535
Furfural ^1^	189	2.2862	***	0.584 ± 0.121	0.141 ± 0.054
*β*-Damascenone ^1^	517	2.163	***	5.126 ± 2.206	1.538 ± 0.55
Isoamyl acetate ^2^	507	2.1451	***	1992.545 ± 536.69	443.673 ± 309.236
Propyl octanoate ^2^	756	1.9695	-	0.576 ± 0.18	0.558 ± 0.37
Hexyl acetate ^2^	654	1.8633	***	75.28 ± 34.752	8.898 ± 17.274
Vinyl decanoate ^2^	786	1.8341	***	42.054 ± 32.322	2.746 ± 3.368
*α*-Ionene ^2^	785	1.7883	***	0.001 ± 0.001	0 ± 0
2-Phenethyl acetate ^2^	483	1.7725	***	57.768 ± 13.622	20.634 ± 11.516
Isobutanol ^2^	167	1.7563	***	31,002.732 ± 7642.192	48,972.568 ± 11,124.274
1-Hexanol ^1^	368	1.6953	**	1787.951 ± 559.742	2611.528 ± 584.55
1-Heptanol ^1^	12	1.6619	***	0.05 ± 0.018	0.388 ± 0.252
Methyl octanoate ^2^	35	1.6531	***	1.711 ± 0.397	3.768 ± 1.234
Ethyl heptanoate ^2^	17	1.6307	***	0.333 ± 0.024	1.178 ± 0.435
Ethyl nonanoate ^2^	34	1.5589	***	1.352 ± 0.151	2.903 ± 0.985
Propane, 1,1,3-triethoxy- ^3^	972	1.5478	***	0.535 ± 0.572	0.072 ± 0.064
Ethyl butanoate ^2^	275	1.5156	***	397.005 ± 25.995	239.21 ± 73.425
Ethyl salicylate ^2^	107	1.5099	***	1.065 ± 0.502	3.017 ± 1.2
1-Nonanol ^1^	43	1.4691	***	0.296 ± 0.188	3.879 ± 1.979
TDN ^2^	931	1.3725	***	8.283 ± 5.478	3.508 ± 1.531
Methyl decanoate ^2^	57	1.368	-	25.455 ± 2.78	35.997 ± 14.337
*α*-Methylnaphthalene ^3^	88	1.3644	**	0.00106 ± 0.000223	0.00225 ± 0.00104
Ethyl butyl succinate ^2^	90	1.3313	**	8.623 ± 7.038	56.841 ± 38.987
(*S*)-3-Ethyl-4-methylpentanol ^2^	137	1.2702	**	21.537 ± 15.378	105.636 ± 63.778
Nonanal ^1^	111	1.2432	-	0.01 ± 0.005	0.021 ± 0.02
Diethyl succinate ^1^	105	1.2407	**	707.764 ± 754.868	6357.521 ± 4451.953
Benzaldehyde ^1^	182	1.1356	-	65.154 ± 59.562	118.818 ± 80.712
1-Decanol ^2^	1025	1.1257	***	10.803 ± 6.564	5.159 ± 2.351
(*E*)-3-Hexen-1-ol ^2^	601	1.1189	*	44.742 ± 34.777	80.819 ± 41.385
Linalool ^1^	1307	1.0883	*	17.954 ± 16.594	6.174 ± 6.813
Isopentyl hexanoate ^2^	253	1.0096	-	6.391 ± 0.867	9.36 ± 4.226

‘*’, ‘**’, and ‘***’ indicate significant differences at *p* < 0.05, *p* < 0.01, and *p* < 0.001, respectively, by Duncan’s multiple-range test; ‘-’ indicates no significant difference. ^1^ Quantified through the standard curves of standards; ^2^ semi-quantified using standards with similar chemical structures; ^3^ quantified by multiplying peak area/internal standard peak area by 1000.

**Table 3:** Mean concentration (ug/L) and standard error of mean (SE mean) of brandy volatiles (VIP > 1) from Yantai, Shacheng, and Tianjin.

Compound	Feature ID	VIP Score	Yantai	Shacheng	Tianjin
(*E*)-Calamenene ^3^	828	2.1026	9.79 ± 1.79 a	2.43 ± 0.73 b	11.36 ± 3.5 a
*α*-Terpineol ^1^	321	2.039	5.69 ± 0.81 b	2.94 ± 1.17 c	8.12 ± 2.35 a
Isopentanol ^1^	292	2.0126	145,840.94 ± 11,667.12 b	218,955.23 ± 34,414.72 a	234,182.48 ± 45,982.5 a
Ethyl lactate ^2^	194	1.9334	25,559.98 ± 4127.49 a	17,195.94 ± 6531.74 b	28,964.51 ± 12,227.76 a
Isoamyl lactate ^2^	208	1.8654	4.11 ± 0.39 a	2.73 ± 0.55 b	3.96 ± 1.38 a
*β*-Damascenone ^1^	375	1.7845	5.15 ± 0.31 c	6.99 ± 0.87 b	7.8 ± 0.79 a
Benzaldehyde ^1^	626	1.7371	32.13 ± 10 b	37.16 ± 22.16 ab	46.43 ± 17.83 a
TDN ^2^	826	1.6852	12.35 ± 2.08 b	15.63 ± 9.56 b	34.92 ± 7.69 a
Methyl decanoate ^2^	444	1.6666	399.98 ± 1.52 c	427.75 ± 15.68 a	413.82 ± 5.52 b
Isobutanol ^2^	163	1.6226	89,967.2 ± 18,784.52 b	116,229.41 ± 29,243.81 a	127,043.67 ± 32,501.45 a
Isoamyl hexanoate ^2^	241	1.622	4.94 ± 1.11 b	15.72 ± 7.68 a	18.49 ± 5.11 a
Isobutyl octanoate ^2^	240	1.6134	1.47 ± 0.55 c	4.68 ± 4.97 b	9.9 ± 3.6 a
1-Propanol ^2^	42	1.5665	17,093.15 ± 3865.7 b	25,809.17 ± 7220.59 a	16,193.9 ± 3949.2 b
*α*-Calacorene ^3^	377	1.5016	6.36 ± 2.69 b	0.87 ± 0.45 c	10.03 ± 6.76 a
Phenethyl acetate ^2^	624	1.4641	43.46 ± 16.3 a	13.87 ± 4.32 b	56.47 ± 52.28 a
Linalool ^1^	409	1.448	6.34 ± 1.65	6.62 ± 2.91	5.19 ± 1.93
Propyl octanoate ^2^	780	1.4393	10.41 ± 5.37 b	67.34 ± 57.43 a	26.63 ± 13.74 b
(*E*)-3-Hexen-1-ol ^2^	353	1.3865	41.12 ± 63.89 b	112.95 ± 69.26 a	7.39 ± 20.1 b
Ethyl 2-hexenoate ^2^	581	1.3563	26.51 ± 1.7 b	26.34 ± 3.5 b	36.29 ± 8.34 a
Isoamyl acetate ^2^	150	1.2785	1048.62 ± 701.74	848.76 ± 268.75	1123.94 ± 490.86
Ethyl decanoate ^2^	282	1.2544	1799.75 ± 503.38 c	6517.48 ± 3360.27 b	8082.6 ± 1807.78 a
Nonanal ^1^	304	1.1166	0.97 ± 1.12 ab	1.59 ± 1.14 a	0.9 ± 0.49 b
Ethyl hexadecanoate ^2^	526	1.0707	673.51 ± 275.44 b	1244.49 ± 596.92 a	1259.46 ± 373.08 a
Hexyl acetate ^2^	305	1.0102	55.82 ± 74.26	42.91 ± 34.8	77.56 ± 97.16

Different letters in the same row indicate significant differences at *p* < 0.05 by Duncan’s multiple-range test; ‘-’ indicates no significant difference. ^1^ Quantified through the standard curve of standard; ^2^ semi-quantified using standard with similar chemical structure; ^3^ quantified by multiplying peak area/internal standard peak area by 1000.

## Data Availability

The original contributions presented in this study are included in the article and the Supplementary Materials. Further inquiries can be directed to the corresponding author.

## Supplementary Materials

The following supporting information can be downloaded at https://www.mdpi.com/article/10.3390/foods13121922/s1: Table S1: Sample information; Table S2: Variables for GC-MS peaks in pot still and continuous still; Table S3: Variables for GC-MS peaks in Yantai, Tianjin, and Shacheng; Table S4: Compounds identified in pot still and continuous still; Table S5: Compounds identified in Shacheng, Tianjin, and Yantai.
